# The Hour of Our Greatest Need

**Published:** 1900-04

**Authors:** 


### THE HOUR OF OUR GREATEST NEED.

The signal victory won in the Senate last week in carrying through our army bill giving rank and a just power to the veterinarian was a true evidence of how far public opinion may be roused in the support of a meritorious measure. There were no party lines drawn on this appeal, but that which was best for our country, our army, our soldiers, won in this final battle in the upper house.

But this great victory in the Senate must be followed by a greater effort in the House of Representatives. Every day means one of earnest work. Every member of the House must be seen and appealed to. Every friend of the measure must give his support now. Every school, association, and organization of veterinarians must command an unalterable support. See at once your Congressman with others, and have every member of the lower branch of our national legislative body urged to support this measure, and the long, earnest battle of ten years will be won triumphantly, and the entire country will rejoice with us in this added measure of security.

